# Glucose-6-Phosphate Dehydrogenase Modulates *Shiraia* Hypocrellin A Biosynthesis Through ROS/NO Signaling in Response to Bamboo Polysaccharide Elicitation

**DOI:** 10.3390/molecules30204060

**Published:** 2025-10-11

**Authors:** Xinping Li, Qunyan Huang, Yanjun Ma, Liping Zheng, Jianwen Wang

**Affiliations:** 1 College of Pharmaceutical Sciences, Dali University, Dali 671000, China; xinping_lee@126.com; 2 College of Pharmaceutical Sciences, Soochow University, Suzhou 215123, China; qyhuang2021@stu.suda.edu.cn (Q.H.); mayjdyx@nwnu.edu.cn (Y.M.); 3 Department of Horticultural Sciences, Soochow University, Suzhou 215123, China; lpzheng@suda.edu.cn

**Keywords:** glucose-6-phosphate dehydrogenase, *Shiraia*, hypocrellin A, bamboo polysaccharides, reactive oxygen species, nitric oxide

## Abstract

Hypocrellin A (HA), a photodynamic perylenequinone pigment from *Shiraia* fruiting bodies, functions as an efficient photosensitizer for clinical photodynamic therapy. Glucose-6-phosphate dehydrogenase (G6PDH), the rate-limiting enzyme of the pentose phosphate pathway (PPP), governs carbon flux into NADPH production. This study elucidates G6PDH’s regulatory role in HA biosynthesis in *Shiraia* sp. S9. Bamboo polysaccharide (BPS) elicitation (100 mg/L) significantly enhanced HA production to 428.1 mg/L, 1.6-fold higher than controls after 5 days. We cloned the *G6PDH* gene and demonstrated that BPS upregulated its expression and activity, concomitant with increased reactive oxygen species (ROS; H_2_O_2_ and O_2_^•−^) and nitric oxide (NO) generation. ROS production was mediated by NADPH oxidase induction, while NO generation was attributed to elevated nitric oxide synthase and nitrate reductase activities. Critically, the G6PDH inhibitor glucosamine (1.0 mM) suppressed both H_2_O_2_ and NO production. These ROS/NO signals upregulated key HA biosynthetic (*PKS*, *Omef*) and transport (*MFS*) genes. Our findings establish G6PDH as a central regulator of BPS-induced HA biosynthesis via ROS/NO signaling, revealing novel metabolic crosstalk between the PPP and fungal perylenequinone biosynthesis. This work presents BPS elicitation as a biotechnological strategy for scalable HA production in *Shiraia* mycelium cultures.

## 1. Introduction

**Figure 1:**
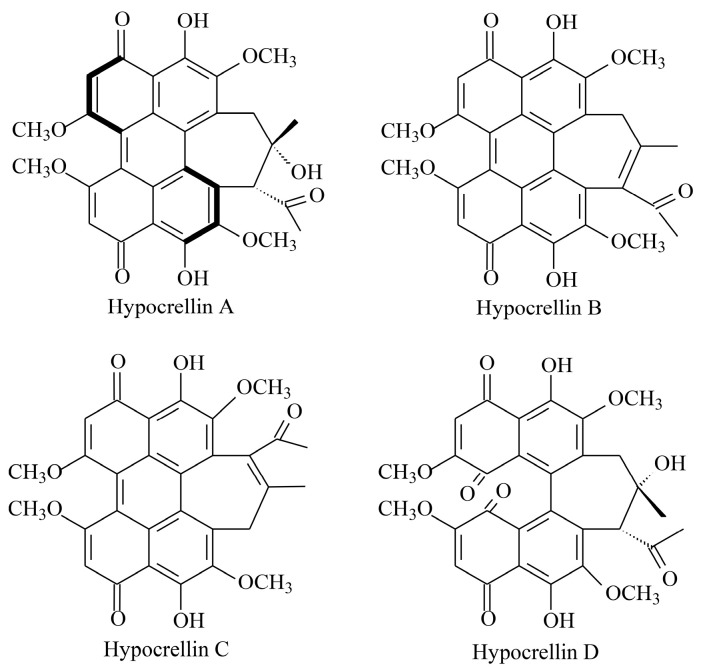
The chemical structures of hypocrellins.

*Shiraia* fruiting bodies, traditionally known as “Zhu Huang” in Chinese folk medicine, have long been used to promote blood circulation, resolve blood stasis, and alleviate rheumatoid arthritis symptoms [1]. These fungi produce bioactive perylenequinone derivatives, primarily hypocrellins A–D (HA–HD) [2] (Figure 1). Hypocrellins exhibit high yields of reactive oxygen species (ROS), particularly singlet oxygen (^1^O_2_), alongside low dark toxicity and rapid clearance, making them clinically valuable in photodynamic therapy (PDT) for vulvar white lesions and lichen amyloidosis [3]. HA demonstrated notable photocytotoxicity by triggering apoptotic cell death in prostate, ovarian, breast, and myeloid leukemia cells [4]. It suppressed tumor proliferation by downregulating fibroblast growth factor receptor 1 (FGFR1) phosphorylation or activating the ROS-mediated NF-κB pathway, positioning HA as a promising PDT photosensitizer for cancer treatment [5]. HA also exhibited potent light-induced antimicrobial activity against drug-resistant pathogens, including azole-resistant *Candida albicans* and methicillin-resistant *Staphylococcus aureus* [6,7]. Furthermore, it inhibited SARS-CoV-2 infection (EC_50_ = 0.22 μM), acting as a potential viral entry inhibitor [8]. The multi-target action of HA-PDT reduced microbial resistance development, highlighting its potential as an alternative to conventional antibiotics. In the food industry, HA is increasingly used as a natural edible colorant and preservative due to its strong dye affinity, lipid solubility, and antimicrobial properties [9,10]. However, HA production remains constrained by the unsuccessful cultivation of *Shiraia* fruiting bodies and the complexity of chemical synthesis [11]. Consequently, HA relies on extraction from wild *Shiraia* fruiting bodies, which are seasonally harvested in degraded bamboo forests of southern China [12], creating a bottleneck for broader applications. *Shiraia* mycelium culture has emerged as a biotechnological alternative for HA production [13]. Methods for solid-state and submerged fermentation have been established, with optimization of inoculum levels, moisture content, pH, medium composition, and incubation time [14,15,16]. Despite these advances, HA yields remain low (e.g., 2.02 mg/g DW in solid-state culture; 10–40 mg/L in submerged culture) [14,17]. Notably, some strains (e.g., *Shiraia* sp. SUPER-H168 and *S. bambusicola* S8) fail to produce hypocrellins in liquid cultures [18,19]. To address this, biotechnological strategies have employed abiotic elicitors (e.g., Triton X-100, ultrasound, light, heat stress, heavy metal ions) and biotic elicitors (e.g., fungal polysaccharides, *Phytophthora boehmeriae* proteins, bacterial cells) to enhance HA production [13]. Elicitation activated defense responses and HA biosynthetic genes via signaling cascades. Transient ROS generation (e.g., H_2_O_2_, O_2_^•−^) was an early oxidative burst that induced HA biosynthesis [20,21], observed in *Shiraia* cultures treated with ultrasound, light/dark shifts, bamboo charcoal, or lanthanum ions [13,22]. Recently, nitric oxide (NO) as a signaling molecule was also found to be involved in elicitor-induced HA production in *Shiraia* mycelium cultures [13,23,24], whereas NO donor sodium nitroprusside was shown to enhance HA yields [25]. NO generation triggered by L-arginine, heat stress, red light, or lipopolysaccharides further stimulated HA biosynthesis [13,23,24]. Crucially, ROS and NO signaling jointly regulate HA synthesis [26,27]. However, no enzyme coordinating both signals in *Shiraia* has been identified, representing a critical gap in understanding HA regulation.

In plants, glucose-6-phosphate dehydrogenase (G6PDH) modulates ROS and NO signaling under saline-alkaline, aluminum, drought, and heat stress [28]. G6PDH catalyzes the first step of the pentose phosphate pathway (PPP), oxidizing glucose-6-phosphate to 6-phosphogluconolactone while producing NADPH—a key source of reducing power [29]. Cytosolic NADPH mitigates ROS damage via the glutathione (GSH)-ascorbate cycle. Stress-induced G6PDH activity maintained redox homeostasis by boosting NADPH and reducing ROS, as demonstrated in *Arabidopsis thaliana* under salt stress [30] and highland barley under alkaline stress [31]. NO, acting downstream of ROS, upregulated cytosolic G6PDH activity in soybeans under aluminum toxicity or drought [32,33] and in red kidney bean roots under salt stress [34]. Conversely, G6PDH inhibition suppressed nitrate reductase (NR)-dependent NO production, indicating G6PDH-NR crosstalk in plant stress tolerance. Paradoxically, G6PDH-derived NADPH fueled NADPH oxidase (NOX)-driven ROS in animal pathologies (e.g., atherosclerosis, heart failure) [35,36,37].

The role of G6PDH in fungal metabolism is less understood. *Saccharomyces cerevisiae zwf1*Δ mutants (lacking *G6PDH*) exhibited oxidant susceptibility and growth defects under vanillin-induced oxidative stress [38,39]. In *Hansenula mrakii*, tert-butyl hydroperoxide induced G6PDH to replenish NADPH for glutathione reductase activity [40]. *Rhizopus nigricans* engineered to overexpress G6PDH enhanced P450-dependent hydroxylation of progesterone [41]. Despite these insights, G6PDH’s regulation of fungal secondary metabolism—particularly via ROS/NO signaling—remains unexplored. Recently, we found that a bamboo polysaccharide (BPS) elicitor (MW: 34.2 kDa; arabinose:galactose = 53.7:36.9) significantly enhanced HA production in *Shiraia* [42]. Building on this, we investigated G6PDH’s role in HA biosynthesis. We optimized BPS elicitation, characterized the *Shiraia* G6PDH gene, and analyzed HA biosynthetic gene expression. We further examined BPS-induced ROS/NO generation and their interplay with G6PDH and HA biosynthesis.

## 2. Results

### 2.1. Eliciting Effects of BPS on Fungal HA Production and G6PDH Activity

**Figure 2:**
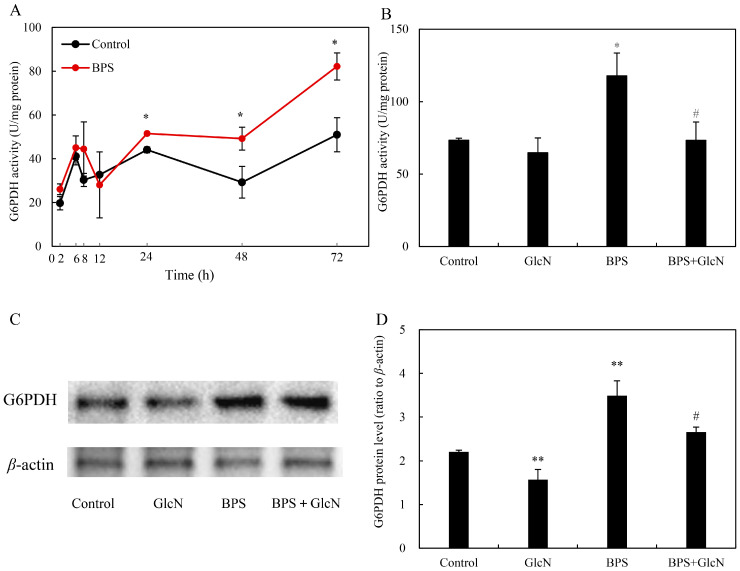
Effect of BPS on G6PDH activity in *Shiraia* sp. S9 mycelia. (**A**) Time profile of the G6PDH activity under BPS treatment. The BPS (100 mg/L) was added on day 3 of the culture. The G6PDH activity (**B**), G6PDH expression (**C**) and its relative protein level (**D**) in *Shiraia* sp. S9 mycelia treated with glucosamine (GlcN) and BPS. GlcN (1.0 mM) was added 30 min prior to BPS treatment. The culture without BPS and GlcN treatment was used as control. The fungal mycelia were cultured at 150 rpm and 28 °C for 6 days. Values are mean ± SD from three independent experiments (* *p* < 0.05 and ** *p* < 0.01 vs. control, ^#^ *p* < 0.05 vs. BPS group).

BPS elicitation (60–200 mg/L) significantly enhanced HA production in *Shiraia* sp. S9 mycelium cultures (Table 1) when added on day 3, whereas higher HA yield (354.8 mg/L) was observed at 100 mg/L BPS. To compare the effect of different addition time, BPS at 100 mg/L was added during the culture from day 1 to day 7, respectively. After 8-day-old culture, both HA biosynthesis and secretion were stimulated only under BPS treatment at 100 mg/L added on day 2 or day 3 (Table 2). Total HA production peaked (428.1 mg/L, 1.6-fold vs. control) only when BPS (100 mg/L) was added on day 3. During the cultures under BPS treatment, there were no significant changes in fungal biomass (Figure S1A), whereas the content of residual sugar in medium decreased markedly (Figure S1B), suggesting the eliciting role of BPS on fungal utilization of carbon sources. Concurrently, BPS treatment (100 mg/L, day 3) markedly stimulated G6PDH activity (82.2 U/mg protein at 72 h; 1.6-fold vs. control; Figure 2A). Pretreatment with the G6PDH inhibitor glucosamine (GlcN, 1.0 mM) suppressed BPS-induced G6PDH activity by 37.9% (Figure 2B). Western blot analysis confirmed reduced G6PDH protein levels under GlcN cotreatment (Figure 2C,D).

### 2.2. G6PDH Cloning and Expression Under BPS Treatment

**Figure 3:**
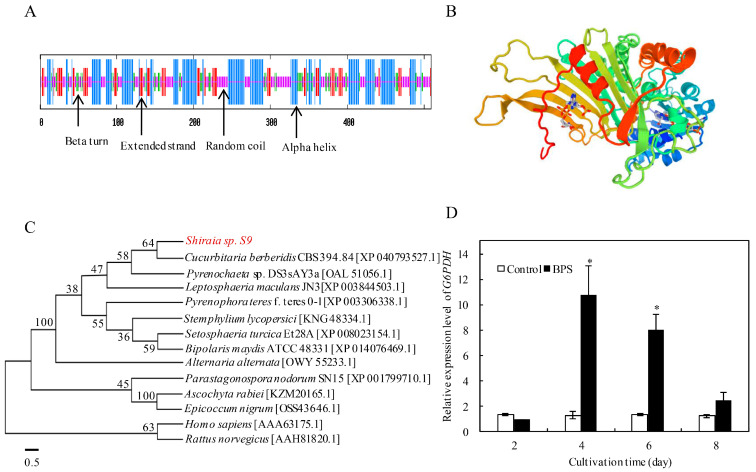
The protein secondary structure (**A**), tertiary structure (**B**) and phylogenetic tree (**C**) of G6PDH in *Shiraia* sp. S9. The expression profile of *G6PDH* under BPS treatment (**D**). The BPS (100 mg/L) was added on day 3 of the culture which was maintained at 150 rpm and 28 °C. The culture without BPS treatment was used as the control (* *p* < 0.05 vs. control group).

The full-length *G6PDH* cDNA (1530 bp ORF, Figure S2) encoded a 509-amino-acid protein (predicted MW: 58.7 kDa; pI: 6.63) with secondary structure comprising 40.5% α-helices, 14.9% β-strands, 39.1% random coils, and 5.5% β-turns (Figure 3A). The protein lacked transmembrane domains and signal peptides (Figure S3A,B), and its predicted tertiary structure conserved NAD^+^-binding and C-terminal catalytic domains (Figure 3B). Phylogenetic analysis placed *Shiraia G6PDH* within the fungal *G6PDH* superfamily, sharing 97.6% sequence similarity with *Cucurbitaria berberidis* CBS 394.84 (Figure 3C), and multiple sequence alignment confirmed high conservation across fungi (Figure S4). BPS treatment significantly upregulated *G6PDH* expression (10.9-fold at 24 h; 8.0-fold at 72 h vs. control; Figure 3D).

### 2.3. Effect of G6PDH on BPS-Induced HA Production

**Figure 4:**
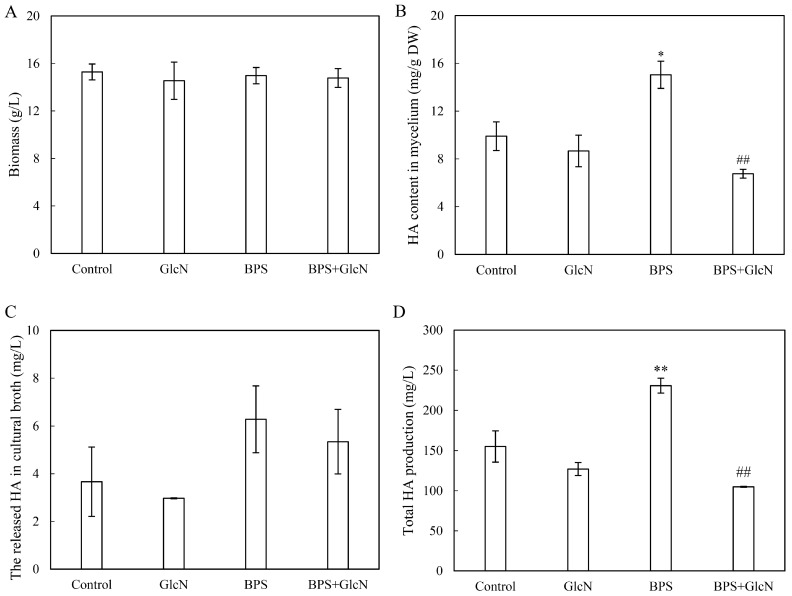
Effects of G6PDH on the growth and HA production of *Shiraia* sp. S9 under BPS treatment. Effects on fungal biomass (**A**), HA content in the mycelium (**B**), the released HA in the culture broth (**C**) and total HA production (**D**). BPS (100 mg/L) was added on day 3 of the culture. Glucosamine (GlcN, 1.0 mM) was added 30 min prior to BPS treatment. The culture without BPS and GlcN treatment was used as the control group. The culture was maintained at 150 rpm and 28 °C and harvested on day 8. Values are mean ± SD from three independent experiments (* *p* < 0.05 and ** *p* < 0.01 vs. control, ^##^ *p* < 0.01 vs. BPS group).

To reveal the mediation of G6PDH in BPS-induced HA biosynthesis, the HA content in fungal mycelia treated with BPS, G6PDH inhibitor GlcN, and the combination of BPS and GlcN (BPS + GlcN) were analyzed. GlcN pretreatment did not alter fungal biomass (Figure 4A), but drastically reduced BPS-induced HA content in mycelia (6.8 mg/g DW; 55.1% decrease vs. control; Figure 4B). Although there was no effect on HA secretion to the medium (Figure 4C), total HA production was decreased by 54.5% to 104.9 mg/L under GlcN pretreatment after BPS elicitation (Figure 4D), demonstrating G6PDH’s essential role in BPS-induced HA biosynthesis.

### 2.4. Effect of G6PDH on BPS-Induced ROS Production

**Figure 5:**
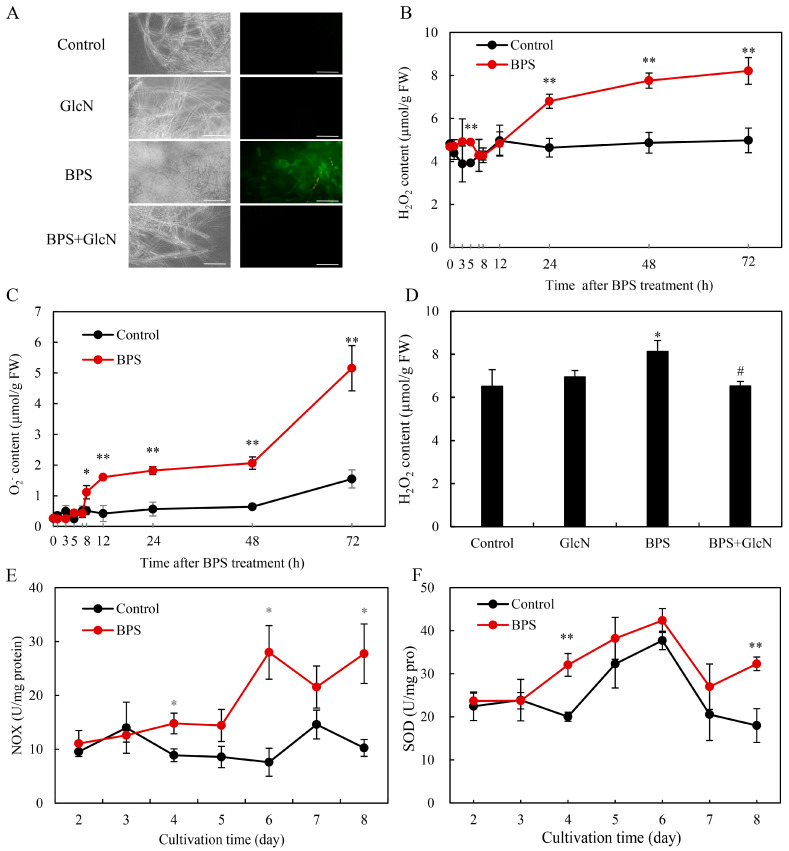
BPS treatment induced ROS generation in *Shiraia* sp. S9. (**A**) Bright-field images (left) and fluorescence microscopy (right) of DCFH-DA-stained mycelia. Scale bars = 50 μm. The H_2_O_2_ content (**B**) and O_2_^•−^ content (**C**) in mycelium after BPS treatment. BPS (100 mg/L) was added on day 3 of the culture. The effect of GlcN on BPS-induced H_2_O_2_ production (**D**). Time profile of the BPS-induced NADPH oxidase (NOX) activity (**E**) and superoxide dismutase (SOD) activity (**F**). Glucosamine (GlcN, 1.0 mM) was added 30 min prior to BPS treatment. The culture without BPS treatment was used as the control. Values are mean ± SD from three independent experiments (* *p* < 0.05 and ** *p* < 0.01 vs. control, ^#^ *p* < 0.05 vs. BPS group).

To further explore the effects of G6PDH on ROS production under BPS treatment, the fluorescent probe 2, 7-dichlorodihydro fluorescein diacetate (DCFH-DA) was used to assess the intracellular ROS level in *Shiraia* sp. S9. BPS-treated mycelia exhibited intensified DCFH-DA fluorescence (Figure 5A) and elevated H_2_O_2_/O_2_^•−^ levels (Figure 5B,C). GlcN cotreatment reduced fluorescence intensity and H_2_O_2_ content by 19.7% (Figure 5A,D), indicating G6PDH’s involvement in ROS generation. BPS also increased NADPH oxidase (NOX) activity, peaking at 28.0 U/mg protein on day 6 (Figure 5E), and enhanced superoxide dismutase (SOD) activity by 12.3–79.6% from days 4–8 (Figure 5F).

### 2.5. Effect of G6PDH on BPS-Induced NO Production

**Figure 6:**
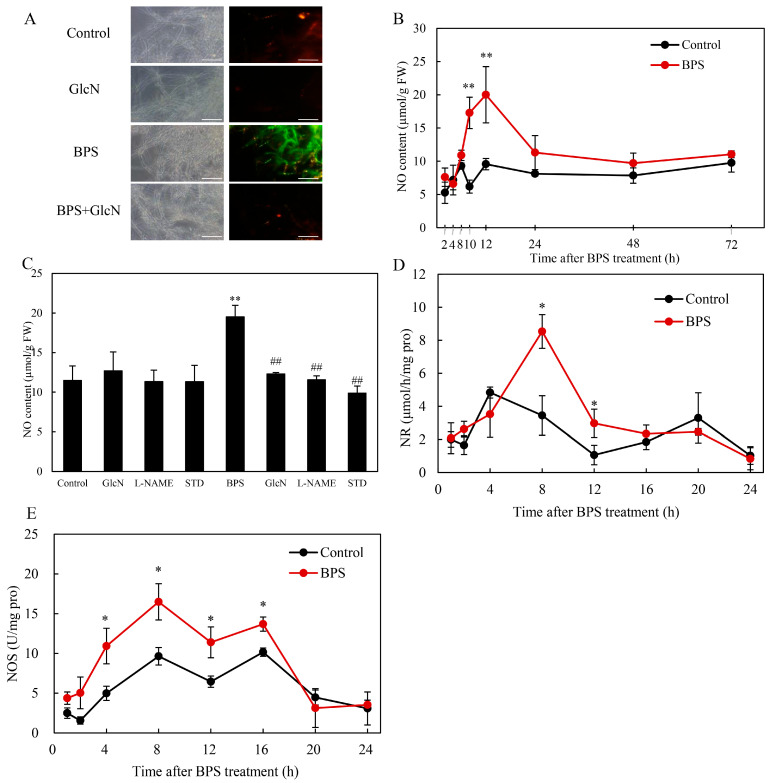
BPS treatment induced nitric oxide generation in *Shiraia* sp. S9. (**A**) Bright-field images (left) and fluorescence microscopy (right) of DAF-2DA-stained mycelia. Scale bars = 50 μm. Time course of NO generation in fungal mycelium (**B**) and the effects of related enzyme inhibitors on NO generation (**C**). The BPS (100 mg/L) was added on day 3 of the culture. Glucosamine (GlcN, 1.0 mM), Nω-nitro-L-arginine methyl ester (L-NAME, 100 μM) and sodium tungstate dihydrate (STD, 100 μM), were added 30 min prior to BPS treatment. The culture without BPS treatment was used as the control. Time profile of the BPS-induced nitrate reductase (NR) (**D**) and nitric oxide synthase (NOS) activity (**E**). Values are mean ± SD from three independent experiments (* *p* < 0.05 and ** *p* < 0.01 vs. control, ^##^ *p* < 0.01 vs. BPS group).

BPS elicited stronger 4-amino-5-methylamino-2′-7′-difluorofluorescein diacetate (DAF-FM DA) fluorescence (Figure 6A) and higher NO content, peaking at 20.0 μmol/g fresh weight (FW) at 12 h (Figure 6B). GlcN reduced NO levels by 36.9% (Figure 6C), confirming G6PDH’s role in NO generation. BPS upregulated nitrate reductase (NR; 8.5 μmol/h/mg protein at 8 h; 2.5-fold *vs.* control; Figure 6D) and nitric oxide synthase (NOS) activity (peak: 16.5 U/mg protein at 8 h; 34.7–119.9% increase; Figure 6E). NOS inhibitor *N_ω_*-nitro-L-arginine methyl ester (L-NAME) and nitrate reductase (NR) inhibitor sodium tungstate dehydrate (STD) decreased NO content by 40.7% and 49.3%, respectively (Figure 6C).

### 2.6. Effect of G6PDH on BPS-Induced HA Biosynthetic Gene Expression

**Figure 7:**
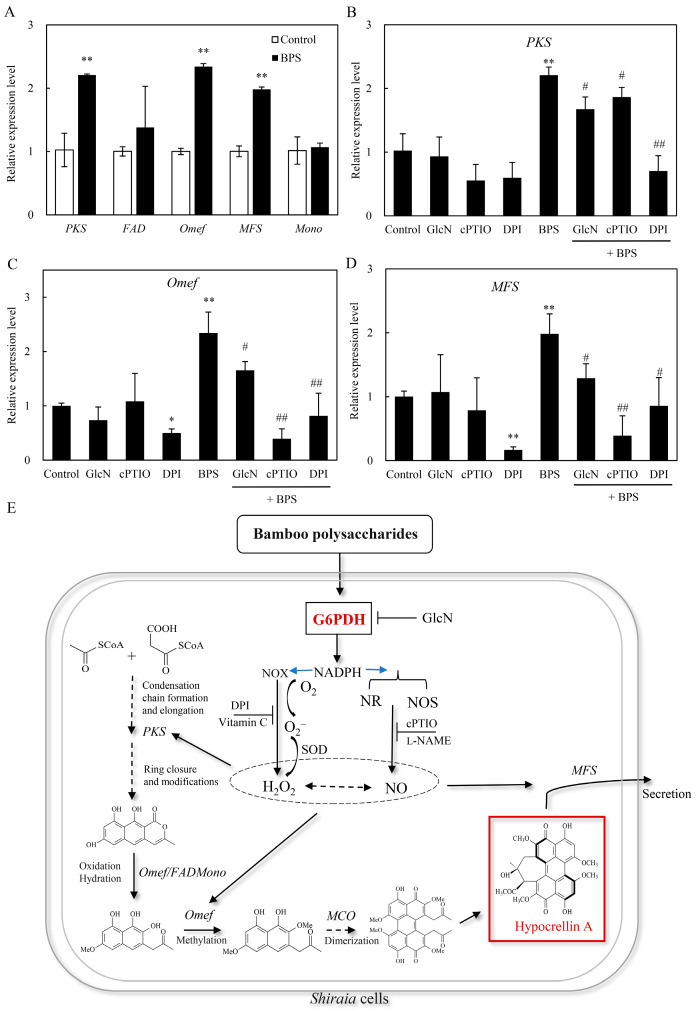
The effect of G6PDH on HA biosynthesis in *Shiraia* sp. S9 induced by BPS treatment. (**A**) Effect of BPS on the gene expressions for *Shiraia* HA biosynthesis. The BPS (100 mg/L) was added on day 3 of the culture. Effect of BPS-induced NO and ROS on the expression of *PKS* (**B**), *Omef* (**C**) and *MFS* (**D**). GlcN (1.0 mM), cPTIO (100 μM), and DPI (5 μM) were added to the culture 30 min prior to the BPS treatment at 100 mg/L. The culture without BPS treatment was used as the control. (**E**) Schematic diagram of the signal pathway for HA biosynthesis and transport in *Shiraia* sp. S9 induced by BPS. The solid arrows indicate promotion of pathway or expression, and lines with blocked ends indicate the inhibition of pathway or expression. Values are mean ± SD from three independent experiments. (* *p* < 0.05 and ** *p* < 0.01 vs. control, ^#^ *p* < 0.05 and ^##^ *p* < 0.05 vs. BPS group).

To investigate whether BPS-induced G6PDH affects fungal secondary metabolism, the expression levels of five HA biosynthetic genes, including polyketide synthase (*PKS*), FAD/FMN-dependent oxidoreductase (*FAD*), *O*-methyltransferase (*Omef*), monooxygenase (*Mono*) and major facilitator superfamily transporter (*MFS*), were detected after 24 h of BPS treatment. BPS upregulated key HA biosynthetic gene expressions (*PKS*: 2.2-fold, *Omef*: 2.3-fold, *MFS*: 2.0-fold vs. control in Figure 7A). This induction was suppressed by GlcN (G6PDH inhibitor), cPTIO (NO scavenger), and DPI (NOX inhibitor), reducing *PKS*, *Omef*, and *MFS* expression by 15.6–68.1%, 29.4–83.2%, and 35.0–80.3%, respectively (Figure 7B–D), highlighting G6PDH-ROS/NO crosstalk in HA biosynthesis.

## 3. Discussion

HA, a photoactivated perylenequinone toxin produced by *Shiraia* species, contributes to the pathogenesis in over ten bamboo species by generating ROS that disrupt host cell integrity and facilitate wound infection [43,44]. Plant cell walls—composed primarily of polysaccharides—serve as colonization sites for pathogenic fungi, and their components can act as signals to stimulate toxin biosynthesis. For instance, amylopectin induced *Fusarium* fumonisin B1 [45], while oligosaccharides trigger *Alternaria* AP-toxin production [46]. Building on our prior observation that bamboo charcoal enhanced HA biosynthesis [22], this study demonstrated that BPS not only elevated intracellular HA but also promoted its secretion into the medium (Table 1 and Table 2). This suggests that *Shiraia* fungi, upon encountering bamboo cell walls, upregulate HA synthesis to photodynamically damage host tissues—a dynamic interplay reflecting host–pathogen coevolution. Genomic and transcriptomic analyses revealed that HA biosynthesis originates from acetyl-CoA and malonyl-CoA condensation via a polyketide synthase (PKS), with precursors supplied by glycolysis and the tricarboxylic acid (TCA) cycle [47,48]. Elicitors like urea and lipopolysaccharides enhanced HA yields by upregulating glycolytic/TCA genes, thereby increasing acetyl-CoA flux [24,49]. Here, BPS (100 mg/L) increased both G6PDH activity and HA production by ~1.6-fold, while the G6PDH inhibitor GlcN suppressed these effects (Table 1 and Table 2; Figure 2A and Figure 4). Our results demonstrated that GlcN not only inhibited the activity of G6PDH but also suppressed its expression (Figure 2). While the suppression of G6PDH enzymatic activity by GlcN is primarily attributed to substrate competition and alterations in the cellular redox state, GlcN treatment could reduce G6PDH mRNA or protein levels in some specific cell lines such as chondrocytes, pancreatic β-cells, and endometrial stromal cells [50]. This downregulation led to a consequent decrease in PPP activity. We propose that GlcN metabolism induces cellular stress in these sensitive cell types, which may trigger a broad reprogramming of gene expression, including the transcriptional regulation of PPP-related genes such as G6PDH. Our study establishes a direct link between G6PDH, the rate-limiting enzyme of PPP and HA biosynthesis, highlighting PPP’s previously unrecognized role in fungal perylenequinone metabolism. Critically, G6PDH likely bridges central carbon metabolism (embden-meyerhof-parnas, EMP/TCA/PPP) with stress-induced HA synthesis through NADPH-dependent ROS/NO signaling (Figure 5 and Figure 6).

Unlike its antioxidant role in yeasts [39,40], *Shiraia* G6PDH stimulated ROS generation by fueling NOX activity (Figure 5). NOX uses cytosolic NADPH to reduce O_2_ to O_2_^•−^, implicating G6PDH-derived NADPH in ROS amplification—a mechanism observed in cardiovascular pathologies [35], aluminum-stressed soybean roots [51], and *Nicotiana benthamiana* hypersensitive responses [52]. As HA’s photodynamic action (type I/II reactions) generates cytotoxic ROS [2], *Shiraia* faces intrinsic oxidative stress during HA production. Other photosensitizer-yielding *Cercospora* species could develop complex strategies include toxin export, transient reduction and quenching by pyridoxine [44]. In our previous study, both HA export and reductive detoxification of HA in *Shiraia* sp. S9 were conducted with the help of *Bacillus cereus* No. 1 [53]. *Shiraia* sp. SUPER-H168 enhanced significantly the activity of antioxidant enzymes including catalase, glutathione reductase, and SOD to 20 mM H_2_O_2_ treatment [20]. BPS-induced G6PDH upregulation (Figure 2 and Figure 3) supplied NADPH to sustain NOX-driven ROS, while parallel SOD activation (12.3–79.6%; Figure 5F) mitigated collateral damage. Unlike plants, where G6PDH-derived NADPH primarily supports antioxidant systems [28], *Shiraia* repurposes it for *pro*-oxidative HA biosynthesis—a unique adaptation warranting further study.

BPS also elevated NO levels (Figure 6A,B), consistent with NO’s role in HA regulation under various elicitation, including L-arginine supplement, heat stress, red light exposure and bacterial lipopolysaccharide elicitation [13]. GlcN suppression of NO accumulation (Figure 6C) revealed G6PDH as a novel regulator of fungal NO biosynthesis. While NO arises from NOS in animals and NOS-like enzymes/NR in plants [54], NOS-like activity was previously shown to produce L-arginine-dependent NO in *Shiraia* [24]. This study also showed the significant contribution of NR in the NO production (Figure 6D,E). G6PDH may enhance NO production by supplying NADPH to NOS (as in vascular endothelium) [55] or by inducing NR (as in salt-stressed plants) [34]. Thus, G6PDH integrated ROS and NO signaling in *Shiraia*’s response to BPS (Figure 7).

Key HA biosynthetic genes (*PKS*, *Omef*, *MFS*) were upregulated by BPS (Figure 7A), aligning with their roles in backbone assembly (PKS/Omef) and toxin export (MFS) [56]. Among them, PKS was for the condensation of acetyl-CoA and malonyl-CoA, and Omef for methylation for the HA backbone formation [57], whereas MFS transporter was shown to export HA from *Shiraia* mycelia and involved in the self-protection against phototoxicity of HA [56]. Suppression of this induction by diphenylene iodonium (DPI, NOX inhibitor), 2-(4-carboxyphenyl)-4,4,5,5-tetramethylimidazoline-1-oxyl-3-oxide (cPTIO, NO scavenger), and GlcN (Figure 7B–D) confirmed G6PDH-ROS/NO crosstalk in regulating HA synthesis and secretion. Optimized BPS elicitation (100 mg/L on day 3) boosted HA titers to 428.1 mg/L, 1.6-fold over controls, surpassing yields from *Shiraia* cultures under the elicitation of ultrasound, light/dark shifts, or red light [13]. Unlike physical elicitors such as ultrasound or specific light regimes [13,23], which require capital-intensive, energy-consuming equipment and complex process control to ensure uniform application in large-scale bioreactors, BPS is a crude extract derived from abundant, renewable bamboo biomass. Its preparation via hot-water extraction and ethanol precipitation is a simple, low-energy, and cost-effective process, making it significantly cheaper than purified biotic elicitors like bacterial lipopolysaccharides or fungal proteins [24]. Furthermore, as a water-soluble compound, BPS integrates seamlessly into standard submerged fermentation processes, ensuring homogeneous distribution and predictable elicitation without the mass transfer limitations associated with solid additives like bamboo charcoal [22]. Its biological stability allows for easy long-term storage and standardized dosage. Therefore, BPS elicitation presents a compelling, industrially viable strategy for the enhanced bioproduction of HA, combining operational simplicity, low cost, high efficacy, and excellent scalability for potential large-scale applications.

## 4. Materials and Methods

### 4.1. Strain and Culture Conditions

*Shiraia* sp. S9 (registered in China General Microbiological Culture Collection Center as CGMCC 16369), isolated from fresh fruiting bodies [25], was maintained on potato dextrose agar (PDA) slants at 4 °C. For HA production, mycelial cultures were initiated by incubating the fungus on PDA at 28 °C for 8 days. HA production used medium components and culture conditions as previously described [23]. For preculture preparation, 50 mL of modified liquid medium (100 g/L potato, 20 g/L starch, 4 g/L NaNO_3_, 1.5 g/L KH_2_PO_4_, 0.5 g/L CaCO_3_ and 0.01 g/L thiamine, pH 6.3) was inoculated with 1 × 10^7^ spores/mL. The preculture was incubated at 28 °C for 2 days with shaking at 150 rpm and then transferred (1 mL) into a 150-mL flask containing 50 mL of the same liquid medium at 150 rpm and 28 °C for 8 days. All BPS elicitation experiments employed 150-mL flasks containing 50 mL of production medium, incubated at 150 rpm and 28 °C. To determine the optimal BPS dosage, BPS at 60–200 mg/L were added on day 3. To assess stage-dependent effects, 100 mg/L BPS was added to cultures from day 1 to 7.

### 4.2. Preparation of BPS

Crude polysaccharides were extracted from dried bamboo shoots [58]. Powdered shoots were extracted in water (1:30 *w*/*v*) at 80 °C for 2 h, filtered (membrane filter, Dongkang, Tianjin, China), and concentrated to 1/30 volume by rotary evaporation (60 °C, reduced pressure). Polysaccharides were precipitated with three volumes of 95% (*v*/*v*) ethanol at 4 °C overnight, centrifuged at 7000 rpm for 15 min (Allegra X-64R, Beckman Coulter, San Francisco, CA, USA), and deproteinized using the Sevag method [59]. The resulting BPS elicitor was stored at −20 °C until use.

### 4.3. Measurement of G6PDH Activity and Expression

BPS-treated mycelia were harvested, washed twice with distilled water, and flash-frozen in liquid nitrogen. Samples (0.2 g) were homogenized in 1.8 mL phosphate buffer (100 μM, pH 7.4) and centrifuged at 4 °C and 4000 rpm for 15 min (Allegra X-64R, Beckman Coulter, San Francisco, CA, USA). The supernatant was used for G6PDH activity assays [60] and immunoblotting. To quantify protein production by Western blotting, 25 μg of total protein was separated by 10% SDS-PAGE, transferred to nitrocellulose membranes (Beyotime Biotech, Shanghai, China), blocked with 5% casein (Beyotime Biotech, Shanghai, China), and probed with rabbit anti-G6PDH antibody (1:1500 dilution; Abcam, Shanghai, China, ab249573). Blots were visualized using horseradish peroxidase-conjugated goat anti-rabbit secondary antibody (1:2000 dilution; CWBIO, Beijing, China, CW0103).

### 4.4. Cloning of Full-Length G6PDH cDNA

Full-length *G6PDH* cDNA was amplified using gene-specific primers (Table S1; Clone1_F/Clone1_R) designed from *S. bambusicola* RNA-seq data (PRJNA323638) [19]. PCR reactions (50 μL) contained: 100 ng cDNA, 0.4 μM primer, 200 μM dNTPs, 1.25 U of Ex Taq polymerase (Takara, Beijing, China), 10 × Ex Taq buffer (5 μL). Cycling conditions: 94 °C for 3 min; 35 cycles of 94 °C (30 s), 55 °C (30 s), 72 °C (120 s); final extension at 72 °C for 5 min. Products were sequenced and assembled using Clone2 primers. 

### 4.5. Measurement of ROS, NO Generation and Enzyme Activities

Intracellular ROS and NO generation in *Shiraia* sp. S9 hyphae were detected using the fluorescent probes DCFH-DA (for ROS) and DAF-FM DA (for NO) (Beyotime Biotech, Shanghai, China), with visualization under fluorescence microscopy (BX51, Olympus, Tokyo, Japan; excitation/emission: 480/520 nm for ROS, 450–490/500–550 nm for NO). H_2_O_2_ and O_2_^•−^ contents were quantified as described previously [25], while total NO levels were measured using a Total Nitric Oxide Assay Kit (Beyotime Biotech, Shanghai, China) following established protocols [27]. Activities of related key enzymes including NOX, SOD, NOS and NR were assayed according to referenced methods [20,24,61]. To investigate the roles of ROS and NO signaling in BPS-induced HA biosynthesis, cultures were co-treated with the NOX inhibitor DPI (5 µM) or the NO scavenger cPTIO (100 μM), based on concentrations validated in prior studies of *Shiraia* [25,27].

### 4.6. Extraction and Quantification of HA

HA was extracted from mycelia and broth as described by Lei et al. (2017) [19]. Quantification used an Agilent 1260 HPLC system with an HC-C18 column (250 × 4.6 mm). HA concentrations were determined against a standard curve generated with purified HA (>98% purity; Chinese National Compound Library, Shanghai, China). Total HA represents the sum of intracellular and extracellular pools.

### 4.7. Quantitative Real-Time PCR (qRT-PCR) 

Total RNA was extracted using an RNAprep Pure Plant Kit (Tiangen, Beijing, China) and the cDNA was obtained using the reverse transcriptase (Fermentas, Burlington, ON, Canada). The primers for the target and internal reference genes were listed in Table S2. The expression levels were measured by qRT-PCR according to our previous report [19], using FastStart Universal SYBR Green Master (Roche, Basel, Switzerland) on a CFX96 Touch Real-Time PCR Detection System (Bio-Rad, Hercules, CA, USA). All reactions were performed under the following conditions: 3 min at 95 °C, followed by 40 cycles of 30 s at 95 °C, 30 s at 56 °C and 15 s at 72 °C. The relative gene expression was calculated from cycle threshold values using the 2^−ΔΔCT^ method.

### 4.8. Statistical Analysis 

The BPS-induced responses were measured in the shake-flask cultures using 150 mL Erlenmeyer flasks containing 50 mL of medium. All experiments were performed in triplicate independent repeats (ten flasks per replicate). Data are expressed as mean ± SD. Significance was assessed using Student’s *t*-test or one-way ANOVA with Dunnett’s post hoc test. A *p*-value < 0.05 was considered statistically significant.

## 5. Conclusions

This study elucidates the regulatory role of G6PDH in BPS-induced signaling pathways that activate HA biosynthesis in *Shiraia* sp. S9. Optimization of BPS elicitation (100 mg/L added on day 3) achieved a high HA yield of 428.1 mg/L. We demonstrated that BPS significantly enhanced G6PDH activity and gene expression, driving intracellular ROS and NO generation and upregulating key HA biosynthetic (*PKS*, *Omef*) and transport (*MFS*) genes. Critically, suppression of HA biosynthesis by the G6PDH inhibitor GlcN and specific ROS/NO scavengers confirmed that G6PDH coupled the PPP via NADPH production to BPS-induced HA biosynthesis. These findings advance our understanding of *Shiraia*–bamboo host interactions through G6PDH-mediated signaling and establish a novel link between PPP in central carbon metabolism and fungal secondary metabolite regulation. This work identifies G6PDH as a promising biotechnological target for enhancing HA production, with engineered *Shiraia* G6PDH-overexpressing strains under combined BPS/ROS/NO elicitation offering significant potential for industrial-scale HA biosynthesis.

## Figures and Tables

**Table 1:** Effects of BPS concentration on the fungal growth and HA production. BPS at 0–200 mg/L was added on day 3 of the culture. The culture was maintained in a 150 mL flask containing 50 mL of the liquid medium at 150 rpm and 28 °C for 8 days. The culture without BPS was used as control. Values are mean ± SD from three independent experiments (* *p* < 0.05 and ** *p* < 0.01 vs. control).

BPS (mg/L)	Biomass (g/L)	HA Content in Mycelium (mg/g DW)	The Released HA in Cultural Broth (mg/L)	Total HA Production (mg/L)
0	7.9 ± 0.8	12.8 ± 0.2	8.3 ± 1.1	109.3 ± 11.7
60	8.2 ± 0.5	19.9 ± 0.7 **	17.9 ± 1.4 **	181.8 ± 16.9 **
100	9.8 ± 1.2	33.6 ± 0.8 **	27.4 ± 2.6 **	354.8 ± 30.7 **
200	9.7 ± 1.2	30.2 ± 0.8 **	17.2 ± 2.0 **	310.2 ± 44.3 *

**Table 2:** Effects of BPS addition time on the fungal growth and HA production. BPS at 100 mg/L was added on different cultural time (1–7 day). The culture was maintained in a 150 mL flask containing 50 mL of the liquid medium at 150 rpm and 28 °C for 8 days. The 8-day-old culture without BPS was used as control. Values are mean ± SD from three independent experiments (* *p* < 0.05 and ** *p* < 0.01 vs. control).

Addition Time (day)	Biomass (g/L)	HA Content in Mycelium (mg/g DW)	The Released HA in Cultural Broth (mg/L)	Total HA Production (mg/L)
Control	15.1 ± 0.3	17.6 ± 1.9	3.4 ± 0.7	269.1 ± 26.6
1	14.6 ± 0.5	19.5 ± 2.9	4.6 ± 1.1	287.6 ± 33.2
2	14.8 ± 0.0	22.2 ± 1.8 *	6.8 ± 0.9 *	334.3 ± 25.3 *
3	15.0 ± 0.4	28.0 ± 3.1 **	9.2 ± 1.0 **	428.1 ± 33.5 **
4	14.7 ± 0.5	20.8 ± 2.7	4.6 ± 0.6	309.2 ± 34.4
5	14.8 ± 0.1	20.4 ± 2.5	3.9 ± 0.5	304.9 ± 38.3
6	14.7 ± 0.7	18.1 ± 2.8	3.3 ± 0.4	267.6 ± 29.4
7	15.4 ± 0.6	16.2 ± 2.3	3.7 ± 1.0	251.4 ± 30.5

## Data Availability

Data are available within the article and Supplementary Materials.

## Supplementary Materials

The following supporting information can be downloaded at: https://www.mdpi.com/article/10.3390/molecules30204060/s1, Figure S1: The time-course of fungal biomass (A) and residual sugar (B) in the mycelium culture of *Shiraia* sp. S9 treated by BPS. BPS (100 mg/L) was added on day 3 of the culture at 150 rpm and 28 °C. The culture without BPS was used as a control. Values are mean ± SD from three independent experiments (* *p* < 0.05, ** *p* < 0.01 vs. control group); Figure S2: The full-length cDNA sequence and deduced amino acid sequence of G6PDH protein in *Shiraia* sp. S9. The start codon (ATG) and the stop box (TAG) are boxed; Figure S3: Predicted (A) signal peptide and (B) transmembrane region of G6PDH protein; Figure S4: Multiple sequence alignment of G6PDH of *Shiraia* sp. S9 with homologous proteins from other fungal G6PDH. Identical residues are in black background, and similar/conserved residues are in gray background; Table S1: Primers sequence for G6PDH clone (F: forward primer, R: reverse primer); Table S2: Primers used in RT-qPCR. F: forward primer, R: reverse primer.
