# Supplementary material for: Glucose-6-Phosphate Dehydrogenase Modulates Shiraia Hypocrellin A Biosynthesis Through ROS/NO Signaling in Response to Bamboo Polysaccharide Elicitation
Source: Molecules. 2025 Oct 11;30(20):4060. doi: 10.3390/molecules30204060 (PMC12566488; doi:10.3390/molecules30204060)
Supplement: Supplementary file 1 [file molecules-30-04060-s001.zip › Electronic supplementary material submit 9-20.pdf]

Electronic supplementary material

Glucose-6-phosphate Dehydrogenase Modulates *Shiraia* Hypocrellin A Biosynthesis through ROS/NO Signaling in Response to Bamboo Polysaccharide Elicitation

Xin Ping Li [1], Qun Yan Huang [2], Yan Jun Ma [2], Li Ping Zheng [3] and Jian Wen Wang [2,*]
*Correspondence: jwwang@suda.edu.cn

Table S1. Primers sequence for G6PDH clone (F: forward primer, R: reverse primer).

Genes	F/R	Sequence(5'-3')
Clone1	F	TCCAGCCAAACGAGAGTGTC
	R	GTTAGTCTCCTTGCGGCCTT
Clone2	F	ACACGACACTCAAACCCACC
	R	AAATCAGAGAATCGCCGCCT

Table S2. Primers used in RT-qPCR. F: forward primer, R: reverse primer.

Gene symbol	Gene name	Sequence
18S	Reference gene	F:GAAAGTTAGGGGATCGAAGA<br>R:TAGTCGGCATAGTTTACGGT
PKS	Polyketide synthase	F:TGCTGAGGTAGCAGTCAAGC<br>R:TTATGCTACGGTCGTCGCTC
FAD	FAD/FMN-containing dehydrogenase	F:TGTGACCGCCATCACCTTAC<br>R:TTGTCGTATGGGTGGGAAGC
MFS	Major facilitator superfamily	F:TCCCGTAGCCTTGCTTTCTG<br>R:CCGGCTTCTTCTTGACGCTA
Omef	O-methyltransferase	F:GAACTACCTGAAGGCACGCT<br>R:GCTCGGAAGGATACTCGCTC
Mono	Salicylate 1-monooxygenase	F:TCTCGGGGAATTATGGCACG<br>R:ACAACCGTTCTCGCATCAGT
G6PDH	Glucose 6-phosphate dehydrogenase	F:GCAGGAAAGGCATAACGAGC<br>R:CAAATCAGAGAATCGCCGCC

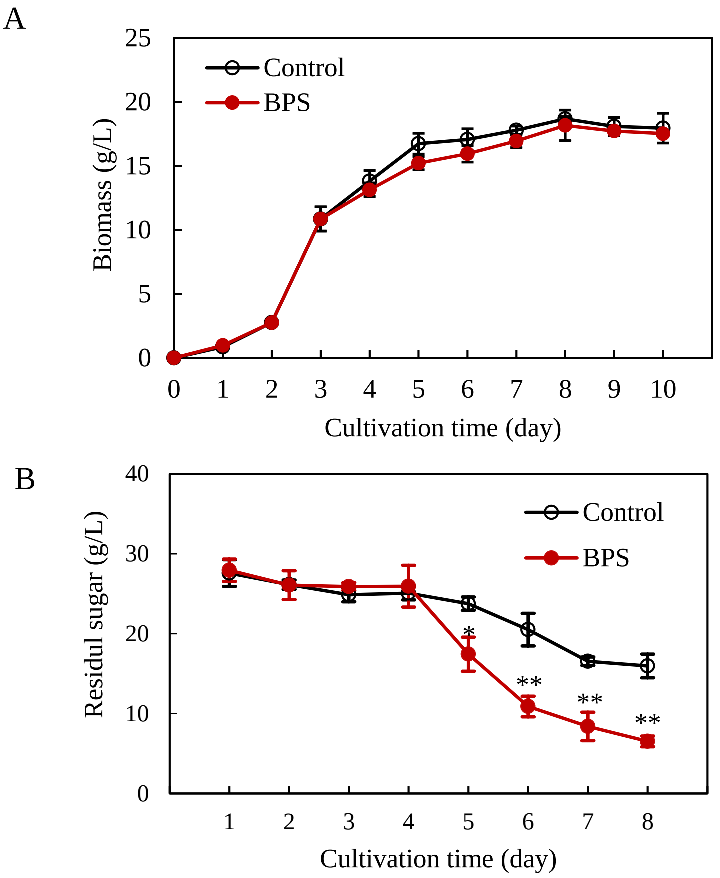

Figure S1. The time-course of fungal biomass (A) and residual sugar (B) in the mycelium culture of *Shiraia* sp. S9 treated by BPS. BPS (100 mg/L) was added on day 3 of the culture at 150 rpm and 28°C. The culture without BPS was used as a control. Values are mean ±SD from three independent experiments (**p*<0.05, ***p*<0.01 vs. control group).

```
1       cggtcagtaagcagtggtatccacgcagagtggccattccggccggccatagctgttttg
61      ccgcttgagctgtcgagaggcgacgtgcacatacgtcaacaccacgatcggcctgccgtg
121     gaccgctaagacgcagggctcccagaccccaccactgaagcttttctttcccaacacgac
181     actcaaacccaccctttcgcacctatcttgagtcgtacggcgctccctggatcctcgttg
241     ttttcgctagcttttaattgagctgaacctatcgaccgttccttccgctttgtgaccagt
301     attgactttatctctgctact
322                          atggccgacaccgtcattcagcctcaacatgaggaggct
                              M  A  D  T  V  I  Q  P  Q  H  E  E  A
361     cacgccggcaacatggagctcaaagacaacaccgtcattatcgttctgggcgcatctggc
         H  A  G  N  M  E  L  K  D  N  T  V  I  I  V  L  G  A  S  G
421     gatctggccaagaagaagactttcccagcgctctttggccttcaccgcaacaacttcctc
         D  L  A  K  K  K  T  F  P  A  L  F  G  L  H  R  N  N  F  L
481     cccaagaacattcgaatcgtcggatatgcaaggacaaagatggaccatgaggagtacctc
         P  K  N  I  R  I  V  G  Y  A  R  T  K  M  D  H  E  E  Y  L
541     aagcgggtcaagtcctatatcaaaacacccaccaaagagcacgagaagcagctagaggag
         K  R  V  K  S  Y  I  K  T  P  T  K  E  H  E  K  Q  L  E  E
601     ttctgtggctactgcacatacgtctcagggcaatatgacaaggatgagtccttccaggag
         F  C  G  Y  C  T  Y  V  S  G  Q  Y  D  K  D  E  S  F  Q  E
661     ctcgagaagcatttgggcgagcttgagaaaggccgcaaggagactaacagaatattctac
         L  E  K  H  L  G  E  L  E  K  G  R  K  E  T  N  R  I  F  Y
721     atggcgctgccacctagtgtcttcatcacagtctctcaacacttgaagcgcaactgctac
         M  A  L  P  P  S  V  F  I  T  V  S  Q  H  L  K  R  N  C  Y
781     ccaaagaacggcatttctcgtgttatcgtcgagaagcctttcggcaaggatctcccaagt
         P  K  N  G  I  S  R  V  I  V  E  K  P  F  G  K  D  L  P  S
841     tcccgagagcttcagcgcgcacttgcacccgactggtctgaggatgagctttaccgcatt
         S  R  E  L  Q  R  A  L  A  P  D  W  S  E  D  E  L  Y  R  I
901     gatcactaccttggcaaagaaatggtcaagaatatcctcattctccgatttggtaatgag
         D  H  Y  L  G  K  E  M  V  K  N  I  L  I  L  R  F  G  N  E
961     tttttcggtgcgacctggaacaggaaccatatcgacaacattcagatcacattcaaagaa
         F  F  G  A  T  W  N  R  N  H  I  D  N  I  Q  I  T  F  K  E
1021    ccattcggtacagagggacgaggtggttactttgatgaatttggcatcatccgtgatgtg
         P  F  G  T  E  G  R  G  G  Y  F  D  E  F  G  I  I  R  D  V
1081    atgcagaatcatttgttgcaggtccttactctcctcgctatggagcgaccgatttctttt
         M  Q  N  H  L  L  Q  V  L  T  L  L  A  M  E  R  P  I  S  F
1141    tctgctgaggatattcgtgacgaaaaggttcgcgtcctccgtggcatggctgccattgag
         S  A  E  D  I  R  D  E  K  V  R  V  L  R  G  M  A  A  I  E
1201    ccaaagaacgtcatcatcggacagtacggcagatctttggatggctcaaagccaggttac
         P  K  N  V  I  I  G  Q  Y  G  R  S  L  D  G  S  K  P  G  Y
1261    aaagaggatgacactgtgcctaaggactcccgatgccccaccttcgcctccatggttgca
         K  E  D  D  T  V  P  K  D  S  R  C  P  T  F  A  S  M  V  A
1321    tatatcaagaacgagaggtgggatggtgtgccattcatcctcaaggcaggaaaggcattg
         Y  I  K  N  E  R  W  D  G  V  P  F  I  L  K  A  G  K  A  L
1381    aacgagcagaagaccgaagttcgcattcaattcaaggatgtcacatcaggtatcttcaag
         N  E  Q  K  T  E  V  R  I  Q  F  K  D  V  T  S  G  I  F  K
1441    gacattccgcgaaacgaattggtcattcgtgtccagccaaacgagagtgtctacatcaag
         D  I  P  R  N  E  L  V  I  R  V  Q  P  N  E  S  V  Y  I  K
1501    atgaactccaagcttcccggtctcagcatgcagacagtcctcacagagctcgatctcact
         M  N  S  K  L  P  G  L  S  M  Q  T  V  L  T  E  L  D  L  T
1561    tacaggcggcgattctctgatttgaagattcccgaggcatacgagtcgctgattctcgac
         Y  R  R  R  F  S  D  L  K  I  P  E  A  Y  E  S  L  I  L  D
1621    gcacttaagggtgaccattccaactttgttcgtgacgatgagctcgatgccagctggagg
         A  L  K  G  D  H  S  N  F  V  R  D  D  E  L  D  A  S  W  R
1681    atcttcactcctctacttcactacctggacgacaacaaggagattattcctatggagtac
         I  F  T  P  L  L  H  Y  L  D  D  N  K  E  I  I  P  M  E  Y
1741    ccctatggatctcgtggccctgctgtcttggacgatttcacctcgtcctacggatacaaa
         P  Y  G  S  R  G  P  A  V  L  D  D  F  T  S  S  Y  G  Y  K
1801    ttcagcgatgcagcaggctaccaatggcccatgaacgagcagaagttgtag
         F  S  D  A  A  G  Y  Q  W  P  M  N  E  Q  K  L  -
1852    agtcgcaatcgcctggaaaagatttggcataatggagtcacacggaaggaaagggaaatg
1912    ctttgctttgattaacgacccgtcagcgaatggatgacgtcagcgaaaggcagacttggg
1972    ccatagataatgaaatcactcccgtctcaaaaaaaaaaaaaaaaaaaaaaaaaaaaaaa
```

Figure S2. The full-length cDNA sequence and deduced amino acid sequence of G6PDH protein in *Shiraia* sp. S9. The start codon (ATG) and the stop box (TAG) are boxed.

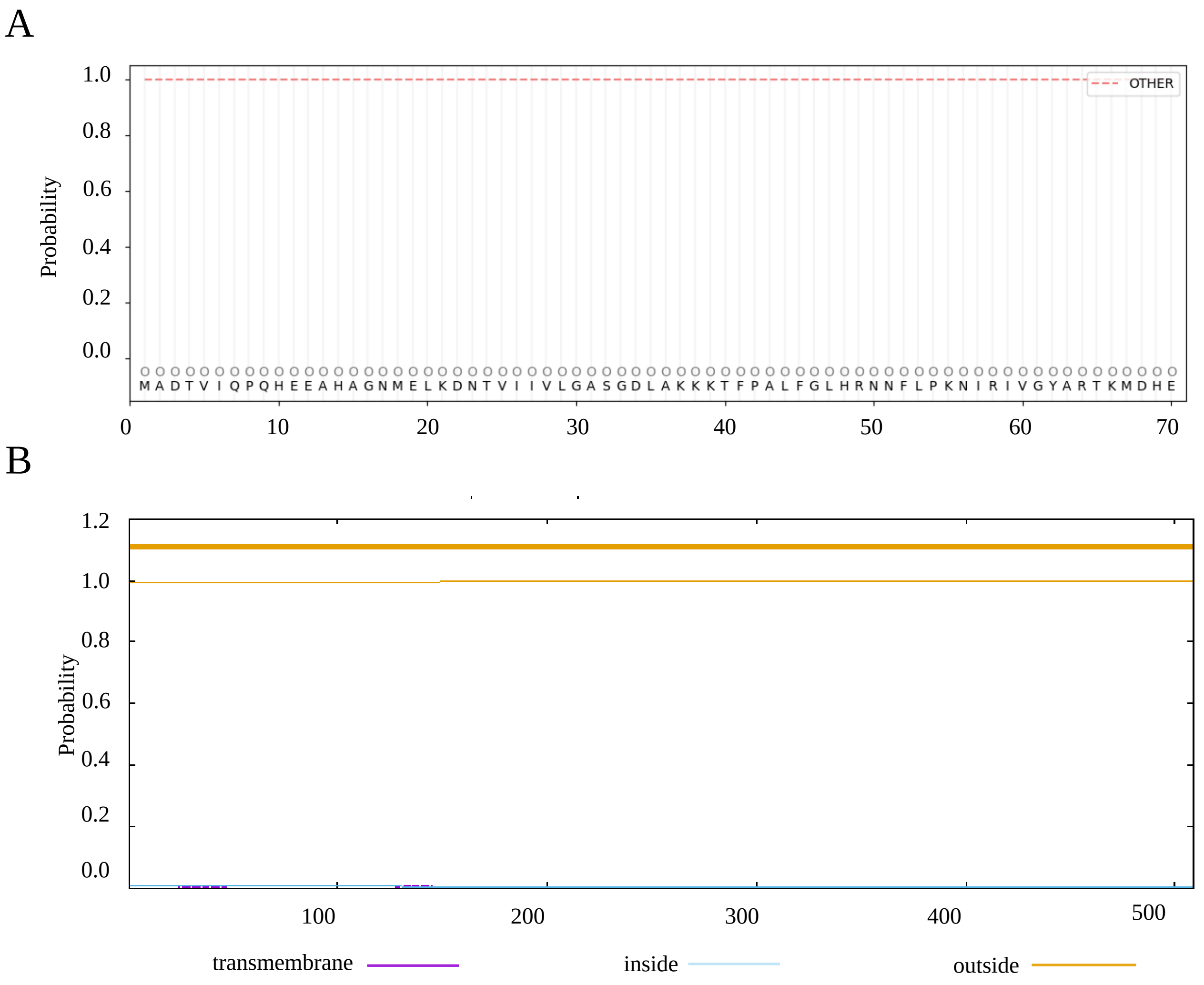

Figure S3. Predicted (A) signal peptide and (B) transmembrane region of G6PDH protein.

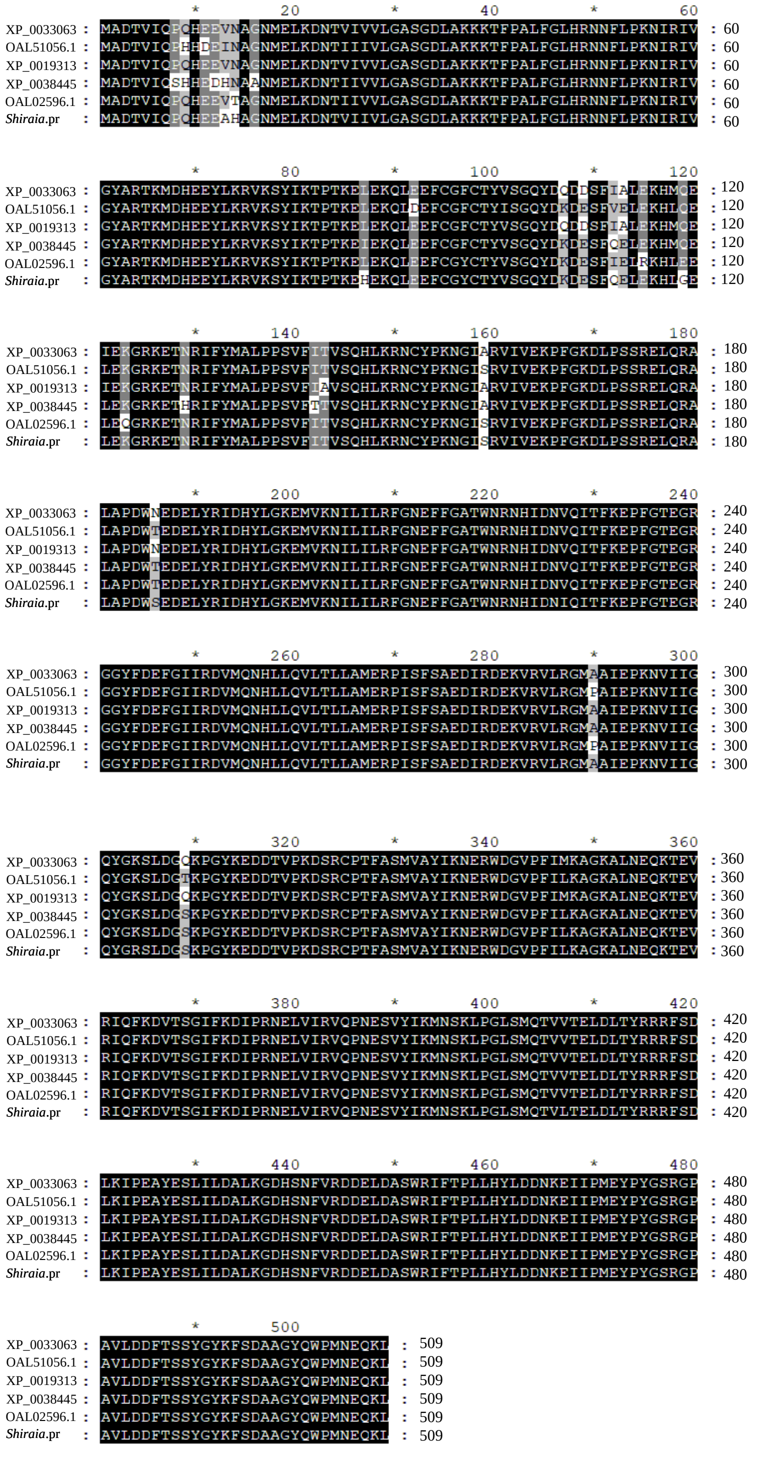

Figure S4. Multiple sequence alignment of G6PDH of *Shiraia* sp. S9 with homologous proteins from other fungal G6PDH. Identical residues are in black background, and similar/conserved residues are in grey background.
